# Views and Needs of Students, Parents, and Teachers on Closed-Circuit Television, Proximity Trackers, and Access Cards to Facilitate COVID-19 Contact Tracing in Schools: Thematic Analysis of Focus Groups and Interviews

**DOI:** 10.2196/44592

**Published:** 2023-05-22

**Authors:** Sofia Chantziara, Ian J Craddock, Claire H Mccallum, Amberly L C Brigden

**Affiliations:** 1 Faculty of Engineering University of Bristol Bristol United Kingdom

**Keywords:** digital contact tracing, school, student, teacher, focus group, proximity tracking, Unified Theory of Technology and Acceptance, UTAUT, acceptance, adoption, CCTV, COVID-19, contact tracing, public health intervention, digital health intervention, implementation, digital tool, technology acceptance, privacy, surveillance technology, surveillance

## Abstract

**Background:**

Contact tracing is considered a key measure in preventing the spread of infectious diseases. Governments around the world adopted contact tracing to limit the spread of COVID-19 in schools. Contact tracing tools utilizing digital technology (eg, GPS chips, Bluetooth radios) can increase efficiency compared to manual methods. However, these technologies can introduce certain privacy challenges in relation to retention, tracking, and the using and sharing of personal data, and little is known about their applicability in schools.

**Objective:**

This is the second of two studies exploring the potential of digital tools and systems to help schools deal with the practical challenges of preventing and coping with an outbreak of COVID-19. The aim was to explore the views, needs, and concerns among secondary school stakeholders (parents, teachers, pupils) regarding the implementation of three digital tools for contact tracing: access cards, proximity tracking, and closed-circuit television (CCTV).

**Methods:**

Focus groups and interviews were conducted with secondary school students, parents, and teachers. The topic guide was informed by the Unified Theory of Technology and Acceptance. Data-driven and theory-driven approaches were combined to identify themes and subthemes.

**Results:**

We recruited 22 participants. Findings showed that there is no single solution that is suitable for all schools, with each technology option having advantages and limitations. Existing school infrastructure (eg, CCTV and smart/access cards technology) and the geography of each school would determine which tools would be optimal for a particular school. Concerns regarding the cost of installing and maintaining equipment were prominent among all groups. Parents and teachers worried about how the application of these solutions will affect students’ right to privacy. Parents also appeared not to have adequate knowledge of the surveillance technologies already available in schools (eg, CCTV). Students, who were mostly aware of the presence of surveillance technologies, were less concerned about any potential threats to their privacy, while they wanted reassurances that any solutions would be used for their intended purposes.

**Conclusions:**

Findings revealed that there is not one tool that would be suitable for every school and the context will determine which tool would be appropriate. This study highlights important ethical issues such as privacy concerns, balancing invasions of privacy against potential benefits, transparency of communication around surveillance technology and data use, and processes of consent. These issues need to be carefully considered when implementing contact tracing technologies in school settings. Communication, transparency, and consent within the school community could lead to acceptance and engagement with the new tools.

## Introduction

School closures have been applied by many governments around the world to temporarily reduce the spread of COVID-19. In the United Kingdom, schools closed the first time between March 18, 2020, and June 1, 2020, and a second time between January 4, 2021, and March 8, 2021 [1]. However, school closures have a negative impact on children’s social, physical, educational, and psychological development, with students from lower-income backgrounds impacted disproportionately [2]. They also affect parents’ ability to work (particularly women’s), resulting in lower productivity and loss of income [2,3].

A key measure in preventing the spread of infectious diseases is contact tracing, which involves identifying people who have been in contact with an infected individual and their subsequent isolation [4]. During the COVID-19 pandemic, governments around the world, including the United Kingdom, adopted a set of measures (ie, social distancing, enhanced cleaning, good ventilation of spaces, use of face coverings, good hand and respiratory hygiene) along with contact tracing and quarantine or testing of close contacts to limit the spread of COVID-19 in schools [5-7].

Contact tracing tools utilizing digital technology can increase efficiency compared to very labor-intensive manual methods [8-10]. Proximity tracking technology includes GPS receivers capable of precise location tracking outdoors, Bluetooth radios that can sense the proximity between devices (usually indoors), and Wi-Fi receivers that give an approximate position by detecting proximity to Wi-Fi access points [8,10]. Mobile phone apps that capture situations where two mobile phones have been in close proximity for a sufficient time for the risk of infection to be high have been developed for digital contact tracing and have been used in several countries around the world [11]. Furthermore, smart cards are also increasingly utilized as identification credentials to control access to certain school areas and monitor attendance [12], and smart card data can be utilized to assist contact tracing efforts [13].

Proximity tracking and smart card technologies can introduce certain privacy challenges in relation to retention, tracking, using, and sharing of personal data [14-17], and little is known about whether stakeholders would find these technologies acceptable for contact tracing in the school setting. Further, little is known about the feasibility of implementing these digital contact tracing tools within a school setting.

Surveillance technologies, ranging from closed-circuit television (CCTV) to biometric technologies [18,19] and systems equipped with facial recognition technology [20,21], are commonplace in UK, US, and Australian schools [18-22]. Previous studies have looked more specifically into the use of CCTV for crime prevention purposes and have found concerns around invasion of privacy, particularly among students, as they believed that cameras were used to monitor their behavior, whereas teachers did not perceive themselves to be the subject of observation [19]. Acceptability and support for CCTV depend on the context such as the location of the cameras, the destination of the data, and the rationale for their use. A balance can, in principle, be struck between the use of CCTV and the impact upon privacy [19].

A previous qualitative study [23] investigated school staff’s view on the potential of digital technologies in supporting contact tracing within the school environment. The study identified three digital solutions that could potentially be used to assist contact tracing in schools: proximity tracking using radiofrequency identification (RFID) cards, CCTV with facial recognition technology, and access cards that would monitor access into specific school areas. The same study [23] highlighted that the priority area for COVID-19 contact tracing technologies is in secondary schools (age range of students 11-18 years). In secondary schools, students have more interactions than in primary schools where students are able to remain largely within class “bubbles.” Therefore, contacts of secondary school students can grow exponentially in the time it takes to conduct manual investigations or if some of the close contacts are missed in this process. Previous studies have indeed shown that there is a greater possibility for larger outbreaks in secondary schools [24,25].

This is the second of two qualitative studies exploring the potential of digital tools and systems to help schools deal with the practical challenges of preventing and coping with an outbreak of COVID-19. The study described herein investigated the views, needs, and concerns among secondary school stakeholders (parents, teachers, and students) regarding the implementation of three digital tools for contact tracing: proximity tracking, CCTV, and access cards. The study was part of the wider Covid-19 Mapping and Mitigation in Schools (CoMMinS) project of the National Institute of Health Research, UK Research and Innovation (R101587-103).

## Methods

### Recruitment

Teachers who were recruited from secondary schools (age range of students 12-18 years) in the wider area of Bristol for the first qualitative study and expressed willingness to participate in the second study were invited. The schools were also asked to send an invitation letter to their parents and students. These were mainstream schools that did not cater exclusively to students with developmental or intellectual disabilities. Convenience and opportunistic sampling methods were also utilized. Researchers used their personal and professional connections to recruit students, parents, and teachers. Eligible secondary school staff were those who held a teaching role, information technology role, or senior/management role in the school (eg, heads and deputy heads), or were a staff member tasked with managing COVID-19 within the school; had access to a video call facility and the internet; and were able to speak English. Secondary school students and parents were eligible if they had access to a video call facility and the internet and were able to speak English. An invitation letter was sent and participants registered their interest using the Research Electronic Data Capture (REDCap) system [26] and provided their contact details.

### Ethical Considerations

Individuals who registered their interest in REDCap were contacted by the first author who ensured that they were provided with and understood all the relevant information about the study. Informed consent in writing was obtained through REDCap from individuals who agreed to participate. Students up to 15 years old were asked to sign an assent form and their parents completed a consent form. Students aged 16 years and above and adult participants signed consent forms. Participants were offered a £20 (~US $25) voucher as recognition for their contribution. All procedures were approved by the Faculty of Life Sciences Research Ethics Committee (reference 112284).

### Data Collection

Data were collected through semistructured focus groups and also through interviews for participants who did not want or could not participate in the focus groups. Interviews and focus groups were conducted via video call, as some infection control measures were still in place in the early phases of this study and this option further facilitated bringing together participants from different locations.

The topic guide (Textbox 1) was informed by the Unified Theory of Technology and Acceptance (UTTA) [27], which was developed to understand technology acceptance,

referring to the adoption and use of technologies for the tasks they were designed to support [28]. The UTTA has integrated elements from eight information technology acceptance models and supports that four constructs influence the intent to use a specific technology: performance expectancy, effort expectancy, social influence, and facilitating conditions. The UTTA model has been used to explore factors that can influence the uptake of contact tracing technologies [29-32] and has been adapted for use in qualitative studies [33]. In addition to the four UTTA constructs, considerations around privacy were also explored as studies have shown that these can affect the uptake of contact tracing tools [17,34].

**Figure 1:**
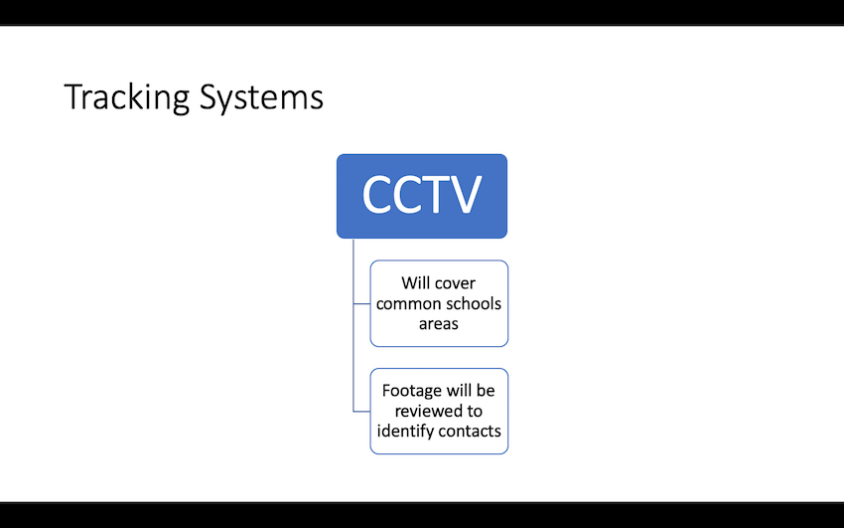
Part of the PowerPoint presentation shown to participants to describe tracking system tools under discussion. CCTV: closed-circuit television.

During focus groups and interviews, participants were shown a PowerPoint presentation that briefly described each tool (Figure 1). The descriptions of the tools were broad and generic as the aim was to elicit views on the acceptability and the potential uses within the school rather than their specific technical characteristics. The descriptions were followed by vignettes explaining how these tools would be applied, for what purpose, who would have access to the data, and for how long the data would be stored (see Textbox 2 for an example vignette).

**Topic guide.**

**Perceived effectiveness (the degree to which an individual believes that using the system will help them perform the required tasks)**

- How effective do you think the system would be in identifying close contacts/increase adherence to social distancing?
- How can the system improve the accuracy and speed of contact tracing?
- How can the system improve adherence to social distancing?

**Effort expectancy** **(the degree of ease associated with use of the system)**

- How easy do you think it would be for you to use the system?
- What would make it difficult for you to use the system?

**Facilitating conditions (the degree to which an individual believes that an organizational and technical infrastructure exists to support use of the system)**

- What support do you require in order to use the system?
- What extra support (eg, infrastructure training) is required for the system to be implemented?

**Social influence (the degree to which an individual perceives that important others believe he or she should use the new system)**

- How might others (eg, teachers, students, depending on group) influence your use of these technologies?
- Who else might encourage or discourage your use of the technology (eg, other people inside or outside schools, wider public, health professionals)? In what circumstances?

**Privacy concerns**

- What are your concerns around sharing your/your children’s personal information?
- How can we address these concerns (eg, changes to the system or wider safeguards in place)?
- Think about your concerns (summarize concerns) and the benefits regarding outbreak control/wider societal benefits (summarize benefits). Would you be willing to accept/use the system?

**Vignette example related to closed-circuit television (CCTV).**

Subject: Use of CCTV in common school areas

Dear Colleagues,

As you know, CCTV is already being used by the school. We are now introducing a new system, equipped with facial recognition technology, that will be in operation in the same common school areas. Footage will be reviewed to identify close contacts of positive cases.

Footage will be stored in school and will only be accessible by the school. Footage will be stored for three months and then would be destroyed.

If you have any thoughts or concerns, please let us know and we’d happy to discuss this with you.

Yours sincerely

Ms Example

Head Teacher

### Analysis

Data-driven and theory-driven approaches were combined to identify themes and subthemes. Themes derived from the UTTA (perceived effectiveness, effort expectancy, context and resources, social influences) informed the theory-driven approach, which included reviewing and revising the themes in context of the data [35]. This was followed by a data-driven approach to expand these predetermined themes and identify subthemes following the principles of thematic analysis outlined by Braun and Clarke [36]. The framework was decided after discussion between two of the researchers considering the relevance of the frameworks to the study. Analysis was facilitated by Nvivo 12 [37].

## Results

### Participants

The study included 6 students (1 female/5 male, age range 12-16 years), 8 parents (7 female/1 male), and 8 teachers (5 male/3 female). Participants were either white British or white other nationality. Teachers were recruited from four secondary schools. Three of the schools were located in the wider area of Bristol and also participated in our preceding qualitative study, and one school was located in Oxfordshire. The size of the schools ranged between 768 to 1012 pupils [38,39].

Students and parents were identified using convenience and snowball sampling after attempts to recruit them from the four participating schools failed. Students and parents were recruited from the southeast, southwest, and southeastern areas of England. Students, parents, and teachers were interviewed separately. Recruitment ended when the research team concluded that any new information would have a minor or no influence on themes that already were emerging from participants’ accounts and it was believed that saturation was reached [40]. Interviews and focus groups lasted on average 30 minutes and they were audio-recorded with participants’ consent. Audio recordings were transcribed and transcripts were pseudonymized.

### Themes

#### Overview

Three main themes were identified: *perceived effectiveness, context and resources,* and *privacy considerations*. The first two themes broadly correspond to UTTA constructs, although they were adjusted to more closely reflect participants’ views and experiences. *Perceived effectiveness* refers to participants’ views on the capacity of the tools to increase the accuracy of contact tracing, potential uses beyond the pandemic, and the factors that can limit their effectiveness. *Contexts and resources* incorporates the UTTA constructs of efforts expectancy and facilitating conditions, and describes views on the burden that each solution could place on students and staff, considerations of the context in each school, and the need for additional resources. We also developed a theme outside the UTTA framework, *privacy considerations,* which describes privacy concerns that need to be addressed to increase acceptance among the school community. The construct *social influences* has been woven into *privacy considerations* as data did not support its existence as an independent theme. The overall themes and subthemes are summarized in Table 1.

**Table 1:** Representative quotes for themes and subthemes.

Themes and subthemes		Quotes		
		Access cards	Proximity tracking	CCTV^a^
**Perceived effectiveness**				
	Some tools may be effective in increasing the accuracy of contact tracing and can have benefits beyond COVID-19	“I was wanting to say that, that also it doesn’t tell you actually if you have been in contact with somebody, it tells you just if you have been in the same location” [Teacher]	“Yeah. I think in, in theory and in a perfect world the, the cards would probably be the most accurate one because everyone’s carrying around something to track where they are and who they’re next to” [Teacher]	“If it did work I think it would be, it would, could be revolutionary for tracing” [Teacher]
	There are concerns about the technical limitations, environmental constraints, and the way students will use the technology, which can limit the perceived effectiveness	“I think that the access cards, I just think there is a high risk of people forgetting to log it, losing it, I think it could cause problems for the students such as if they forgot their card at home and they couldn’t get into the canteen or something like that so I just think it’s a difficult system to maintain” [Student]	“They can leave it [RFID^b^ card] somewhere and you know, I can just imagine what some of them are going to get up to with that. They can throw it over the walls. They can take somebody else’s and…” [Parent]	“You might not be able to catch all of the people they were next to because there’s only a certain number of CCTV cameras you can have and there’s only, you know, a certain amount of places they can see” [Student]
**Effort expectancy**				
	Utilizing existing resources should be a priority as the cost of installing and replacing equipment could discourage schools from adopting certain digital tools	“In some schools it’s going to be easier to implement than others, you know, for in- like our school we don’t have cards for lunches and that, but other schools do. So that’s where I think it’s going to be more difficult” [Teacher]	“I also think that access cards and proximity tracking will be like expensive because new students come to the school every year and we don’t know how long COVID is going to last so the school has to invest money in those access cards and proximity tracking for a long time” [Student]	“The CCTV one could be a good idea because it could be quite cheap if they’ve already got lots of CCTV” [Student]
	Burden for students and staff should be considered when deciding on the most appropriate tools	“It’s about the cards, it’s very easy to lose and I don’t think that everybody takes care of things unless they leave it at the end of the day at school and then they pick it up the next day and they carry it in school” [Parent]	“And in terms of practicalities, so the students would have to carry it how? How do, because I’m just thinking they don’t remember their normal school equipment, let alone carrying around a card” [Teacher]	“I agree with the CCTV because the proximity tracking, they might use the code or something messing with the beacon so I think the CCTV will be better” [Student]
**Privacy considerations**				
	Clear explanation regarding the use of tools; preference for less invasive technology and emphasis on security features and consensus among the school community are needed to ease privacy concerns	“I think access cards I would feel a lot more comfortable with that because it’s more private and the school don’t need to surveil everything you do every minute so for the students I think it would be more comfortable” [Student]	“You need to, you know, make people feel comfortable that this, any of these systems is used only and just for the purpose of saving lives not, and it’s not going to be used, or it is not going to be usable for any other purpose” [Student]	“I think they would have to be very careful that no information gets leaked or released to anyone else because like for example CCTV footage of students it’s quite private I think not everyone would want that published to everyone” [Student]
	It is very difficult for any authority to guarantee that personal data would not be shared with third parties, while privacy invasions cannot always be justified by public health benefits	“I think the access cards are probably the best one, the first suggestion, but I think the proximity tracking and then the CCTV, it doesn’t sit well with me at all. I don’t like the idea of either of those” [Parent]	“Generally, everything you do in life contains a risk and we are way past that point of a risk reward with any of these proposals here so you know getting in a car is dangerous you know, getting on a plane is dangerous, these are all risks in life” [Parent]	“I’ve not signed up for any of my daughter’s image rights or facial recognition to be then data which data is referred to as the new black gold, this is all very valuable data that is being collected, harvested, and who knows where that will be used in the future?” [Parent]

^a^CCTV: closed-circuit television.

^b^RFID: radiofrequency identification.

#### Theme 1: Perceived Effectiveness

Both proximity tracking and CCTV were considered tools that could increase the accuracy of contact tracing. With proximity tracking, it would be possible to track individuals everywhere they go, while CCTV would recognize every individual in the school. There was also the option—for the schools that had the necessary technical infrastructure—to link proximity tracking cards to their existing systems such as class registers and seating plans, which would enhance the accuracy of contact tracing by making it easier to establish the whereabouts of an individual. Access cards were considered a less effective tool, as they could not identify individuals in close proximity to the positive case.

Parents and teachers also wanted contact tracing data to be used for improving infection control measures within the schools rather than purely for contact tracing. Students also expressed the view that systems could be used beyond the pandemic to protect them from bullying and other violent incidents.

Despite their perceived benefits, all tools had certain limitations. CCTV could not cover all school areas (eg, toilets). The success of proximity tracking depends on students’ behavior and there were concerns that students may forget, lose, or play with their cards. Controlling access to certain school areas could lead to students being locked out if they forget or lose their card or congregating at an access point. The potential of the tools to improve infection control measures, with functions such as proximity warnings sent by proximity tracking cards and use of access cards to control access in overcrowded areas, was also explored initially in the focus groups. However, it became apparent early on during data collection that such functions would not be practical within the school. Space limitations made it impossible for students to keep 2 meters distance from each other. Controlling access to certain school areas could lead to students struggling to access facilities, such as toilets, during the limited breaks. Therefore, the themes focus on the potential of the tools to assist with contact tracing.

#### Theme 2: Context and Resources

All stakeholders raised concerns regarding additional responsibilities from the implementation of these tools for teachers and students and also the need for additional resources from the schools. Students, parents, and teachers were worried that asking students to carry and use access or proximity tracking cards would place additional responsibilities on them as they would need to remember to carry the card and to swipe or tap when entering rooms/areas. Students also showed a preference for CCTV as they did not have to actively engage with the technology. Furthermore, teachers and parents were worried that teachers could be asked to be heavily involved in monitoring data for contact tracing, such as reviewing CCTV footage, and this would increase their existing workload and distract from their teaching duties. Teachers voiced the need to have a designated member of staff responsible for contact tracing.

There were concerns, particularly among teachers but also among parents and students, about the ability of schools to buy, install, and replace equipment. The physical layout of the school (eg, physical barriers between different areas) and existing technology and infrastructure in each school (eg, CCTV or a card system already in operation) influenced participants’ views on the suitability of each tool. Access cards would be more appropriate for schools where staff and students already use cards to enter various school areas and therefore doors and access systems were already in place. As cards were already utilized by some schools for security purposes to monitor who is accessing the buildings, the new system would have a dual purpose, which could increase its acceptability. Similarly, if CCTV was already in operation, it would reduce the need for purchasing and installing cameras.

Teachers and students were also concerned about the costs of replacing equipment. This was particularly relevant to proximity tracking/access cards as these solutions required everyone in the school to carry a card, making it more likely for the cards to need frequent replacement as they could be damaged or lost. Teachers highlighted the need for a wider discussion and agreement within the school regarding who will cover the costs of replacing lost or damaged equipment. One option would be for the teachers or students to pay for replacement and another option would be for the schools to incorporate these additional costs into their yearly budget. Teachers shared their experiences on how they had to provide masks for their students on a daily basis when this was required by official government guidelines, since they were failing to bring their own, and eventually having to charge parents for the additional costs. Holding the schools responsible for any replacements would result in a financial burden, which was considered prohibitive for some of the schools involved in the study.

#### Theme 3: Privacy Considerations

Participants discussed concerns around privacy and how these could be addressed. All stakeholders wanted clear explanations of how the tools would be used, including any potential uses in the future. All stakeholder groups, to some extent, also expressed a preference for less invasive methods that did not capture identifiable personal/biometric data; they favored access cards and proximity tracking over CCTV. For some of the parents and teachers, safeguards, especially for CCTV and facial recognition technology, were not possible. They were concerned that students’ personal information could be shared with third parties, particularly private companies, as they were considered a profitable commodity.

Parents also discussed how they were not always aware of the surveillance tools already used by the schools, particularly CCTV, and felt they had less control over their children’s personal information and how these are collected and shared. Some parents also believed that invasions of privacy were not justified based on public health benefits, as the perceived risks from the virus were not considered severe, particularly for children. Parents and teachers appeared more preoccupied with protecting students’ privacy, while students wanted reassurances that data would be kept within the school and shared only with those necessary for the intended purposes (eg, used for contact tracing and not for monitoring students), and they did not express any additional concerns. Teachers wanted official guidance from the government and input from the scientific community to feel more confident in applying the new tools. Agreement and consent from parents were also important for teachers.

## Discussion

### Principal Findings

#### Rationale

This study investigated the views, needs, and concerns among secondary school stakeholders (ie, parents, teachers, and students) regarding the implementation of three digital tools for contact tracing: proximity tracking, CCTV, and access cards. Although technology has been applied widely to assist contact tracing during the COVID-19 pandemic, little is known about applications within a school setting. Teachers, parents, and students are those who will be asked to use and will be impacted directly by any new technologies. Furthermore, these individuals do not form a homogenous group, but have their own expectations and views regarding the use and application of the various tools. Understanding the needs and concerns of each stakeholder group will facilitate the design of tools that will be more likely to be accepted and implemented.

#### Different Digital Tools For Different Schools

Our findings showed that there is not a single tool that is suitable for all schools as all had their own advantages and limitations. CCTV was viewed as the tool that could potentially identify every individual in the school, therefore increasing the accuracy of contact tracing, and it was favored by students as it would not require their active involvement. However, it was acknowledged that it could not cover all school areas and was also considered as the most invasive option. Participants believed proximity tracking could increase accuracy, as it could track individuals everywhere within the school and it could also provide more anonymity. However, its effectiveness was highly dependent on students carrying and using their cards responsibly, which places additional burden on students and increases the space for mistakes. Access cards were viewed as the least invasive option; however, they were also considered the least accurate and their implementation will depend highly on the geography of the school (eg, barriers between areas that would require students use their access cards to access them). Their use could also create further challenges with the potential to cause congestion and create problems for students/staff who forgot or lost their cards. Participants highlighted that available infrastructure (eg, CCTV already in place, widely used card system, barriers between areas) would also determine which tools can be implemented in each school. Concerns regarding the cost of installing and maintaining equipment were prominent among all groups, while concerns around privacy were more prominent among parents and teachers compared to students.

#### Existing Infrastructure and Perceived Costs Shape Views on the Applicability of Various Tools

Context is an important factor in the adoption of digital innovations [27]. Participants of this study highlighted that existing infrastructure in each school was going to determine which tool was more likely to be implemented due to the perceived costs of installing new technologies. Participants were not provided with a cost assessment for each tool, nor were they encouraged to weigh the potential costs of technology against the potential cost savings from effective contact tracing. The costs of extensive school closures to the whole society are significant and can be long-lasting. During the COVID-19 pandemic, a large number of mothers were taking a leave from work, reducing hours, or exiting the labor market as a result of school closures [41-43]. School closures also have an impact on the educational attainment of children and their future earnings [44,45]. These aspects should be highlighted so that any decisions regarding new technologies do not only focus on the perceived costs for the schools but also consider the wider economic benefits of keeping schools open.

#### Concerns Around Privacy Are More Prominent Among Parents and Teachers and Must Be Addressed Within the School Community

We found that concerns around privacy were more prominent among parents and teachers compared to students. Previous research has investigated the perceptions of students regarding the introduction of CCTV in schools [46]. Although students believed that CCTV affected their privacy and initially opposed this technology, over time it was gradually accepted as part of the school environment [46]. It is possible that students that participated in this study have already accepted the presence of surveillance technologies in their schools and they wanted some additional safeguards to be in place. Previous research has looked into the acceptance of surveillance technologies for crime prevention, whereas we explored acceptance of new technologies for public health purposes. Students may have understood the need for mitigation in the middle of a global pandemic and therefore they were more willing to accept these new technologies.

Furthermore, students do not develop these perceptions in isolation but rather these are part of their overall schooling experience, their perception of their educators, and level of trust within the school [47]. Students were aware of the surveillance technologies in their schools, while parents who appeared more concerned about the new tools had limited knowledge. Since building trust is an important factor in addressing privacy concerns, more transparency is required, particularly between schools and parents. There were also some participants who believed that there can be no guarantees that data will be kept safe. Concerns among users of contact tracing apps that third parties will be accessing their personal data have been highlighted in the related literature [34]. Again, communication with the schools and transparency will help address these fears.

### Strengths and Limitations

This study offers new evidence regarding the applicability of digital contact tracing tools in schools while exploring views and needs among various members of the school community. A strength of the study was the inclusion of all three main stakeholder groups: students, parents, and teachers.

However, the small sample sizes within the stakeholder groups along with recruitment strategies have influenced the generalizability of the results. The teachers in their majority were recruited from one geographical area and also participated in the first of the two studies. Parents and students were identified through convenience sampling. As a result, the sample lacked diversity, limiting the applicability of the results to different settings. The tools were not described in detail, making it difficult for the participants in some cases to understand how these would be deployed.

We believe that this study provides important early data on the acceptability and feasibility of different digital systems. Further studies should be performed “in the wild” to identify optimal solutions and include a more diverse and larger sample enabling the generalizability of results in different contexts.

### Conclusion

Findings revealed that there is not one tool that would be suitable for every school. The context will determine which tools would be appropriate. It is important for schools to be transparent, especially with parents, regarding surveillance technologies already available in schools. This will help to build trust and pave the way for the implementation of new technologies. Communication, transparency, and consent within the school community could minimize concerns and fears, and lead to acceptance and greater engagement with the new tools.

## Abbreviations

CCTV: closed-circuit television
CoMMinS: Covid-19 Mapping and Mitigation in Schools
RFID: radiofrequency identification
REDCap: Research Electronic Data Capture
UTTA: Unified Theory of Technology and Acceptance

## Data Availability

Data supporting the findings of this study available from the first author after request.
