# Correction: Views and Needs of Students, Parents, and Teachers on Closed-Circuit Television, Proximity Trackers, and Access Cards to Facilitate COVID-19 Contact Tracing in Schools: Thematic Analysis of Focus Groups and Interviews

**DOI:** 10.2196/67607

**Published:** 2024-11-05

**Authors:** Sofia Chantziara, Ian J Craddock, Claire H McCallum, Amberly L C Brigden

**Affiliations:** 1 Faculty of Engineering University of Bristol Bristol United Kingdom

In “Views and Needs of Students, Parents, and Teachers on Closed-Circuit Television, Proximity Trackers, and Access Cards to Facilitate COVID-19 Contact Tracing in Schools: Thematic Analysis of Focus Groups and Interviews” (JMIR Form Res 2023;7:e44592), the authors noted one error:

In the originally published manuscript, one of the grant codes in the *Acknowledgements* section was incorrectly listed as follows:

National Institute for Health Research (NIHR) and UK Research and Innovation (UKRI) COVID-19 Rapid Response Initiative (R101587-103)

This has now been corrected to:

National Institute for Health Research (NIHR) and UK Research and Innovation (UKRI) COVID-19 Rapid Response Initiative (MR/V028545/1)

The correction will appear in the online version of the paper on the JMIR Publications website on November 5, 2024, together with the publication of this correction notice. Because this was made after submission to PubMed, PubMed Central, and other full-text repositories, the corrected article has also been resubmitted to those repositories

